# Mitochondrial Dynamics: Functional Link with Apoptosis

**DOI:** 10.1155/2012/821676

**Published:** 2012-03-22

**Authors:** Hidenori Otera, Katsuyoshi Mihara

**Affiliations:** Department of Molecular Biology, Graduate School of Medical Science, Kyushu University, Fukuoka 812-8582, Japan

## Abstract

Mitochondria participate in a variety of physiologic processes, such as ATP production, lipid metabolism, iron-sulfur cluster biogenesis, and calcium buffering. The morphology of mitochondria changes dynamically due to their frequent fusion and division in response to cellular conditions, and these dynamics are an important constituent of apoptosis. The discovery of large GTPase family proteins that regulate mitochondrial dynamics, together with novel insights into the role of mitochondrial fusion and fission in apoptosis, has provided important clues to understanding the molecular mechanisms of cellular apoptosis. In this paper, we briefly summarize current knowledge of the role of mitochondrial dynamics in apoptosis and cell pathophysiology in mammalian cells.

## 1. Introduction

Apoptosis, also called programmed cell death, is a crucial physiologic process in the development and homeostasis of multicellular organisms [1]. Perturbation of this vital process leads to a range of diseases, such as ischemia, cancer, neurodegeneration, and autoimmunity [2]. The mitochondrial outer membrane (MOM) serves to coordinate mitochondrial function with extra mitochondrial signaling and participates in the regulation of mitochondrial homeostasis. Mitochondria have a central role in the initiation of apoptosis triggered by intrinsic death signals such as DNA damage (the mitochondrial pathway) by releasing cytochrome *c* and other apoptogenic factors stored in the intermembrane space (IMS) into the cytoplasm [3, 4]. Cytochrome *c* complexed with Apaf-1 activates caspase 9, which leads to the activation of downstream caspases [5].

 Mitochondrial morphology changes dynamically by continuous fission and fusion to form small units or interconnected mitochondrial networks, and the balance of these dynamic changes is essential for counteracting deleterious mitochondrial processes. Mitochondrial fusion allows for complementation of damaged mitochondrial DNA and other contents (e.g., lipids, proteins, or metabolites) with the components of healthy mitochondria, thus maintaining normal mitochondrial activity. Mitochondrial fission, on the other hand, plays an important role in the quality control of mitochondria, facilitating the removal of damaged mitochondria to maintain cellular homeostasis [6–10]. Compromise of this quality control system induces cell death, which results in various degenerative disorders [9]. Mitochondrial fission is also essential for the distribution of mitochondria in response to the local demand for ATP or calcium buffering [10]. In addition to these fundamental roles, the dynamic morphologic changes of mitochondria are closely associated with the initial process of apoptosis. The rate of fission increases markedly when cells become committed to apoptosis; apoptotic stimuli such as DNA injury, UV radiation, endoplasmic reticulum (ER) stress, oxygen radicals, or cytokine withdrawal trigger extensive mitochondrial fission accompanied by cristae disorganization and permeabilization of the mitochondrial outer membrane (MOMP), which in turn induces the release of IMS-stored proapoptotic factors, such as cytochrome *c*, to trigger the apoptosis program [11–15]. Although modulation of mitochondrial fusion and fission machineries is considered to influence the apoptotic response of the cells, it remains controversial whether fission is absolutely required for the progression of apoptosis. Nonetheless, perturbations of the mitochondrial dynamics cause cellular dysfunction, particularly of highly polarized cells such as neurons, and neuronal synaptic loss and cell death in neurodegenerative disorders (e.g., Alzheimer's disease, Parkinson's disease, and Huntington's disease), although the functional relation between the morphologic alterations and apoptosis is still insufficiently understood [16].

## 2. Regulation and Physiologic Significance of Mitochondrial Fusion and Fission

Three types of high-molecular-weight GTPase proteins regulate mitochondrial fusion and fission in mammals [10]. Outer membrane fusion involves two mitofusin proteins (Mfn1 and Mfn2; Fzo1 in yeast) located on the mitochondrial outer membrane (MOM) [17, 18]. An IMS-localized GTPase, OPA1 (Mgm1 in yeast), functions as a hetero-oligomeric complex of the larger size Opa1 (L-Opa1) and the smaller size Opa1 (S-Opa1) in fusion and cristae organization of the inner membrane (MIM) [19, 20]. The cytoplasmic dynamin-related GTPase protein Drp1 (Dnm1 in yeast) translocates to the foci of future mitochondrial fission sites and mediates mitochondrial fission [21–23]. Mitochondrial fission factor (Mff), and mitochondrial dynamics (Mid) 51/mitochondrial elongation factor 1 (MIEF1), and the variant Mid49 were recently reported to function as Drp1 receptors on the MOM [10, 24–27], although detailed mechanisms of Mff and MiD/MIEF1 proteins and their relation in Drp1-dependent mitochondrial fission remain to be clarified. The function of the mammalian homolog of yeast Fis1, which is thought to regulate mitochondrial fission as in yeast remains controversial [10, 24, 25].

 Mutations in the mitochondrial fusion factors Mfn2 and OPA1 result in neurodegenerative disorders, such as Charcot-Marie-Tooth Neuropathy 2a and Dominant Optic Atrophy I, respectively [16, 19, 20]. Mitochondrial fusion factor knockout (KO) mice are lethal before embryonic day 12.5 (E12.5 for Mfn1 KO) or embryonic day 11.5 (E11.5 for Mfn2 KO), suggesting that both Mfn isoforms are essential for embryonic development in mammals [17]. Cells lacking both Mfn1 and Mfn2 exhibit severe cellular defects, including poor cell growth, heterogeneity of inner membrane potential, and decreased respiration, indicating that mitochondrial fusion has an essential role in maintaining functional mitochondria. Depletion of Mfn2 in neurons in mice leads to highly specific degeneration of Purkinje neurons [17]. The mitochondria in these mutant cells are fragmented and fail to distribute to the long and branched neurites, indicating that fusion also plays an important role in mitochondrial distribution in polarized cells. Depletion of both Mfn isoforms in skeletal muscle results in muscle atrophy [28]. Homozygous mutation of OPA1 in mice leads to embryonic lethality by E13.5 in mice, while heterozygous mutation causes a slow onset of degeneration in the optic nerves [29]. Pancreatic beta-cell-specific OPA1 KO mice have compromised glucose-stimulated insulin secretion and ATP production due to a defect in respiratory complex IV, suggesting that the function of OPA1 in the maintenance of the respiratory chain is physiologically relevant to beta cells [30].

**Figure 1:**
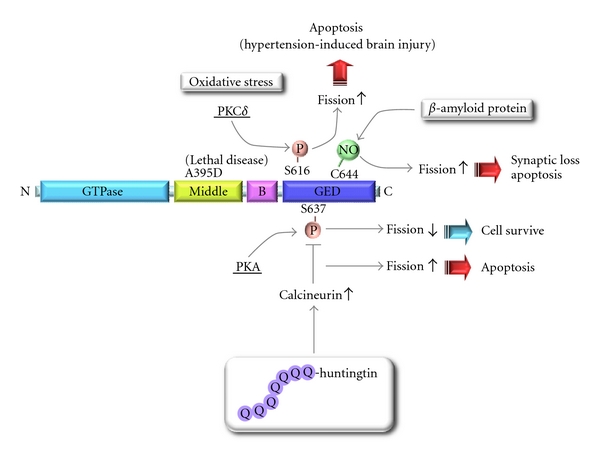
Domain structure of Drp1 and schematic view of the regulation of Drp1 by posttranslational modifications. Drp1 activity is regulated by various posttranslational modifications and changes in these modifications are related to several disorders. Under oxidative stress, protein kinase C*δ* (PKC*δ*) phosphorylates Drp1 at Ser616 in the GED domain. Drp1 is recruited to mitochondria and stimulates mitochondrial fission, leading to apoptosis in hypertension-induced brain. Cyclic-AMP-dependent protein kinase (PKA) phosphorylates Drp1 at Ser637 in the GED domain. This reaction releases Drp1 from mitochondria to the cytosol, leading to mitochondrial elongation and suppression of apoptosis vulnerability of the cells. Calcineurin dephosphorylates Drp1 at Ser637 and promotes mitochondrial fragmentation and cell vulnerability to apoptosis. Polyglutamine expansion in huntingtin protein activates calcineurin and increases mitochondrial fragmentation and cell vulnerability to apoptosis. *β*-Amyloid protein increases S-nitrosylation of Drp1 at Cys644 in the GED domain to trigger mitochondrial fission by activating GTPase, thereby causing synaptic loss. All amino acid numbering is based on the human Drp1 splice variant 1 sequence.

 The dynamin-related GTPase Drp1 localizes mainly in the cytoplasm and plays a central role in mitochondrial fission in mammals. It is composed of an N-terminal GTPase domain thought to provide mechanical force, a dynamin-like middle domain, a connecting domain (“B” in Figure 1), and a C-terminal GTPase effector domain (GED) (Figure 1). Compared with mitochondrial fusion, however, the *in vivo* function of Drp1-dependent mitochondrial fission is poorly understood. During mitochondrial fission, Drp1 existing as small oligomers in the cytoplasm assembles into larger oligomeric structures at the mitochondrial fission sites depending on GTP binding and then severs the mitochondrial membrane by GTP hydrolysis. A heterozygous, dominant-negative mutation of the Drp1 gene (A395D in the middle domain) was identified in a newborn girl with severe pleiotrophic defects, including abnormal brain development and optic atrophy, who died at 37 days of age (Figure 1) [31]. To elucidate the detailed physiologic roles of mitochondrial fission *in vivo*, we and another group generated tissue-specific Drp1 KO mice [32, 33]. Drp1 KO mice die at around E12.5 with developmental abnormalities, particularly in the forebrain. Neuron-specific Drp1 KO mice are born, but die within a day of birth due to neurodegeneration, although Drp1 is dispensable for the viability of mouse embryonic fibroblast (MEF) cells. In primary cultured neural Drp1 KO cells, enlarged mitochondrial clumps are sparsely distributed in the neurites and the synaptic structures are lost. These findings suggest that the Drp1-deficiency causes the abnormal distribution of fused and aggregated mitochondria in polarized cells and these spatiotemporal defects might inhibit the ATP supply and Ca^2+^ signaling, eventually preventing synapse formation. Similarly, Drp1-dependent mitochondrial fission is essential for immune synapse formation in T-cell receptor signaling [34]. A missense mutation in mouse Drp1 in the middle domain, which is essential for oligomerization (Python mice; C452F mutation), leads to cardiomyopathy [35]. The physiologic relevance of Drp1 in other tissues that might underlie various human diseases remains to be elucidated.

 Drp1 activity is regulated by various posttranslational modifications and changes in these modifications are related to several disorders (Figure 1). In the early mitotic phase, Ser616 in human Drp1 is specifically phosphorylated by the Cdk1/cyclinB complex, which promotes mitochondrial fission to facilitate stochastic distribution of the mitochondria to daughter cells [36]. Under oxidative stress conditions, protein kinase C*δ* mediates phosphorylation of Ser579 in human Drp1 isoform 3 (Ser616 in the human Drp1 isoform 1), leading to mitochondrial fission and impaired mitochondrial function, which contributes to hypertension-induced brain injury [37]. Phosphorylation at Ser637 in the GED domain of human Drp1 by cAMP-dependent protein kinase (PKA) stimulates Drp1 GTPase activity and releases Drp1 from the mitochondria by inhibiting oligomeric assembly on the membrane to promote mitochondrial network extension and cell viability [38]. This reaction is reversed by calcineurin-mediated dephosphorylation [39, 40]. Polyglutamine expansions in huntingtin protein, the cause of Huntington's disease, superactivate calcineurin through enhanced calcium levels, and increase mitochondrial recruitment of Drp1, leading to apoptosis due to mitochondrial fission, cristae disintegration, and cytochrome *c* release (Figures 1 and 3) [41]. Further, mutant huntingtin protein directly binds Drp1 and increases its GTPase activity, leading to mitochondrial fragmentation and defects in anterograde and retrograde mitochondrial transport and neuronal cell death [42, 43]. *β*-Amyloid protein, a key mediator of Alzheimer's disease, is reported to induce S-nitrosylation of Drp1 at Cys644 in the GED domain to trigger mitochondrial fission by activating GTPase and thereby causing synaptic loss (Figure 1) [44], although this model has been challenged [45]. Thus, mitochondrial morphologic balance shift toward fission makes cells susceptible to apoptosis and vice versa (Figure 1).

## 3. Regulation of Mitochondrial Apoptosis by Bcl-2 Family Proteins

Mitochondria play a central role in apoptotic initiation by providing proapoptotic factors that are involved in caspase activation, and chromosome condensation and fragmentation [15]. Multiple cellular pathways trigger apoptosis [46]: an extrinsic pathway that is initiated by the binding of a death ligands to the plasma-membrane-localized receptor, resulting in the rapid activation of caspases in the cytoplasm and an intrinsic (mitochondrial) pathway where mitochondria play a central role governed by pro- and antiapoptotic Bcl-2 family proteins. The extrinsic pathway does not directly involve the mitochondria, and activation of the initiator caspase (caspase-8) is mediated by the death-inducing signaling complex [47]. Conversely, the intrinsic pathway is initiated by the release of cytochrome *c* from the IMS accompanied by MOMP and cristae disorganization, which activates procaspase-9 through Apaf-1 [3, 4, 15]. Although the extrinsic and intrinsic pathways have long been considered independent from each other, evidence that caspase-8 also activates the intrinsic pathway has led to a more complex view of apoptosis in which crosstalk exists between the two pathways [48, 49].

 The Bcl-2 family proteins regulate the MOM integrity and contribute to the release of proapoptotic factors from the IMS to the cytoplasm by MOMP [50, 51]. Irrespective of the precise mechanism, the antiapoptotic members of the Bcl-2 family tend to stabilize the barrier function of the MOM, whereas proapoptotic Bcl-2 family proteins such as Bax or Bak tend to antagonize such function and permeabilize the MOM. Upon apoptotic stimuli, caspase-8 activated by the death-inducing signaling complex cleaves the proapoptotic BH3-only protein Bid to the active truncated form (tBid). The activated tBid is then recruited to the cardiolipin-rich region on the MOM by MTCH2, a half-type carrier superfamily protein [52]. tBid either interacts with antiapoptotic Bcl-2 family proteins such as Bcl-2 or Bcl-XL to inhibit their antiapoptotic functions [48, 49, 53], or triggers targeting and oligomerization of cytoplasmic Bax into the MOM and Opa1-dependent cristae disorganization, leading to the release of IMS-stored proapoptotic factors such as cytochrome *c* [54]. Bak, in contrast to Bax, is constitutively localized in the MOM associating with voltage-dependent anion channel 2 (VDAC2) as an inactive form in the ~400-kDa complex, and tBid activates Bak by releasing it from the complex, leading to MOMP [55–58]. Bcl-XL interacts with Bax on the mitochondrial surface and retrotranslocates it to the cytosol, thereby preventing Bax-induced MOMP in healthy cells [59]. As discussed below, mitochondrial membrane dynamics have an important role in the regulation of MOMP and apoptosis.

## 4. The Role of Mitochondrial Fission in Apoptosis

**Figure 2:**
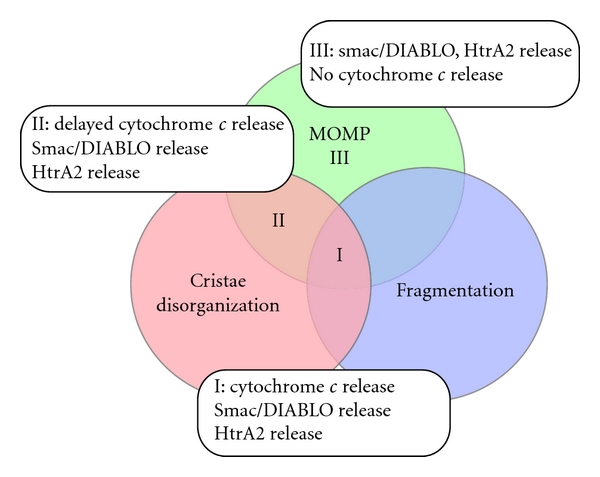
Relation between mitochondrial structural changes and the release of IMS-stored apoptogenic factors. During apoptosis in wild-type cells, mitochondrial fragmentation normally occurs concomitantly with MOMP, cristae disorganization, and subsequent release of the IMS-stored apoptogenic factors (e.g., cytochrome *c*, smac/DIABLO, HtrA2/omi). Mitochondrial fission facilitates these reactions (State I). In contrast, elongated but cristae-disrupted mitochondria (i.e., Drp1- and OPA1-double RNAi cells; Drp1-KO and OPA1 RNAi cells) exhibit a significant delay only in the cytochrome *c* release in response to apoptotic stimuli, because of the absence of mitochondrial fragmentation. Of note, however, MOM targeting and oligomeric assembly of Bax and the release of the IMS-soluble Smac/DIABLO normally proceed (State II). State III: presumed MOM permeabilized state in the absence of both cristae disorganization and mitochondrial fragmentation. MOMP: mitochondrial outer membrane permeabilization.

It is generally accepted that the mitochondrial network collapses into small spherical structures in response to apoptotic stimuli, and that proapoptotic and antiapoptotic Bcl-2 family member proteins play important roles in regulating mitochondrial morphology [15]. During apoptosis, cytosolic Bax is recruited to the MOM and colocalizes with Drp1 and Mfn2 at mitochondrial sites where fission subsequently occurs [60]. Bak, which initially localizes uniformly on the MOM, also coalesces into discrete foci at mitochondrial fission sites during apoptosis. tBid-triggered Bax/Bak activation correlates with a reduction in mitochondrial fusion, possibly through the inhibition of Mfn2 and eventually leads to mitochondrial fragmentation [61, 62]. Upon Bax activation, Drp1 stably associates with the MOM through Bax/Bak-dependent SUMO modifications of Drp1 [63]. This mitochondrial fragmentation is caspase independent and occurs concomitantly with MOMP, cristae disorganization, and subsequent cytochrome *c* release (Figure 2) [64, 65]. Increased mitochondrial fission in apoptotic cells apparently parallels the release of cytochrome *c*, and inhibition of fission by Drp1-RNA interference (RNAi) delays the release of cytochrome *c*, suggesting that the release of cytochrome *c* from the IMS is intimately involved in mitochondrial fission [65]. Consistent with these data, Mff depletion by RNAi results in extensive mitochondrial elongation, delayed cytochrome *c* release, and retardation of apoptosis [24, 66]. Similarly, MEFs from Drp1-KO mice exhibit a delay in cytochrome *c* release, caspase activation and nuclear DNA fragmentation [32, 33]. Notably, mitochondria with network structures that are subtly different from the structures observed prior to cytochrome *c* release are frequently detected in Drp1 KO cells after the release of cytochrome *c* and seem to undergo fragmentation in the advanced stage of apoptosis, suggesting that Drp1-independent mitochondrial fragmentation likely occurs late after the release of cytochrome *c* [32]. This suggests that Drp1-independent fission might participate in mitochondrial fission during apoptosis; for example, *Drosophila* PMI and its human homolog TMEM11 of MIM, both of which regulate mitochondrial morphology in a manner independent of Drp1 and Mfn [67]. Taken together, these findings indicate that the delay in cytochrome *c* release in these cells is relatively modest, suggesting that the Drp1-Mff system is dispensable, but facilitates the normal progression of apoptosis [32, 33]. Conversely, the inhibition of mitochondrial fragmentation by the activation of fusion-related proteins, such as Mfn1, Mfn2, or Opa1 antagonizes apoptosis progression.

 A pharmacologic inhibitor of Drp1-GTPase, mdivi-1, inhibits tBid-dependent cytochrome *c* release from isolated mitochondria that are incapable of undergoing fission in vitro. These findings suggest either that mdivi-1 inhibits other Drp1 functions than mediating mitochondrial fission or that it inhibits molecules other than Drp1 that regulate cytochrome *c* release [68]. Martinou and coworkers recently demonstrated that Drp1 promotes the formation of a nonbilayer hemifission intermediate in which the activated and oligomerized Bax forms a hole, leading to MOMP [69].

 Therefore, although mitochondrial fragmentation is indeed associated with apoptosis, excessive mitochondrial fragmentation can occur in a variety of conditions independently of apoptosis processes, such as that occurring upon exposure to carbonyl cyanide m-chlorophenyl hydrazone (CCCP), uncoupling agents that disrupt the electrochemical potential of the MIM [70]. Thus, how Drp1 contributes to apoptosis is an important issue for future studies.

## 5. Cristae Remodeling and Apoptosis

Opa1, localizing in the inner membrane as a hetero-oligomeric complex of large and small size forms, regulates MIM fusion and is necessary for maintenance of the cristae junctions independently of mitochondrial fusion. The majority of cytochrome *c* is confined within the cristae folds and the complete release and mobilization of cytochrome *c* in the IMS require cristae remodeling or cristae-junction opening [71]. Opa1 depletion by RNAi leads to fragmented mitochondria with disrupted cristae structures and an increase in the sensitivity to the apoptotic stimuli [65, 72, 73]. Further, during early apoptosis, the Opa1 hetero-oligomer is disrupted, the cristae widen, and cytochrome *c* is released into the IMS. Of note, we demonstrated that Opa1 RNAi HeLa cells have disrupted cristae and efficient sensitivity to apoptosis, based on the cytochrome *c* release. In contrast, Opa1/Drp1 or Opa1/Mff double-RNAi cells have elongated but cristae-disrupted mitochondria, and exhibit a significant delay in the cytochrome *c* release in response to apoptotic stimuli (State II in Figure 2) [24]. Importantly, however, mitochondrial targeting of Bax and the release of the IMS-soluble Smac/DIABLO proceeded with the same kinetics as in the control cells. Similarly, in Drp1 KO MEFs or Drp1 RNAi HeLa cells, Bax/Bak-mediated MOMP occurs independently of Drp1 and is separable from cytochrome *c* release. These results suggest that cristae disorganization and mitochondrial fission as well as MOMP (State I in Figure 2) are essentially required for efficient cytochrome *c* release and each can limit the initial apoptosis progression. In contrast to these observations, detailed analysis with transmission electron microscopy and three-dimensional electron microscope tomography revealed that neither cristae reorganization nor cristae-junction opening is required for the complete release of cytochrome *c* [74]. Thus, the requirement of Opa1-dependent cristae remodeling for cytochrome *c* release remains to be reconciled.

## 6. Mitochondrial Morphologic Responses in Cell Survival

Many lines of evidence indicate that the efficiency of oxidative phosphorylation by the mitochondrial electron transport chain is affected by the degree of mitochondrial connectivity; a highly connected mitochondrial network correlates with increased ATP production efficiency. Mitochondria hyperfuse and form a highly interconnected network when cells are exposed to modest levels of stress (e.g., UV irradiation, actinomycin D treatment), named stress-induced mitochondrial hyperfusion (SIMH) [75]. SIMH depends on Mfn1, Opa1, and the MIM protein SLP-2, and delays the activation of Bax and MOMP similar to the beneficial effect of mitofusin overexpression. This seems to be a counterstress action of the cells that is necessary for survival by increased mitochondrial ATP production. Under nutrient starvation, mitochondrial fission is repressed in response to PKA-dependent Drp1 phosphorylation of Drp1 Ser637 due to increased cAMP levels (Figure 1), resulting in elongation of the mitochondria with a higher density of cristae and a capacity for efficient ATP production. This response protects mitochondria from autophagosomal degradation and sustains cell viability [76, 77]. Taken together, enhanced fusion in the mitochondrial fusion/fission balance promotes cell survival. Alternatively, dysfunctional or damaged mitochondria are selectively eliminated by autophagic degradation (termed mitophagy): the process essential for maintaining mitochondrial quality and cell function. For example, accumulation of a causal gene product of Parkinson's disease, PTEN-induced mitochondrial protein kinase 1 (PINK1) on depolarized mitochondria facilitates recruitment of cytoplasmic Parkin, an E3 ubiquitin ligase, to mitochondria to initiate mitophagy [78–80]. The Parkin-PINK1 system thus monitors damaged mitochondria, and dysfunction of this mechanism is a possible cause of inflammation or Parkinson's disease [81–86]. As it is thought that mitochondrial fission is related to the progression of mitophagy, inhibition of mitochondrial fission by the dominant negative mutant of Drp1 or specific inhibitor of Drp1-GTPase mdivi-1 compromises Parkin-PINK1-dependent mitophagy [83]. Together, mitochondrial fusion and fission are more likely to be involved in mitochondrial quality control in healthy cells. A recent report demonstrated that the A-kinase anchoring protein 1 (AKAP1) localized on the MOM is involved in this reaction. Thus, the PKA/AKAP1 complex-calcineurin system regulates mitochondrial morphology and cell viability by controlling the translocation of Drp1 to the mitochondria (Figure 1) [87].

## 7. Communication between the Mitochondria and ER in Apoptosis

**Figure 3:**
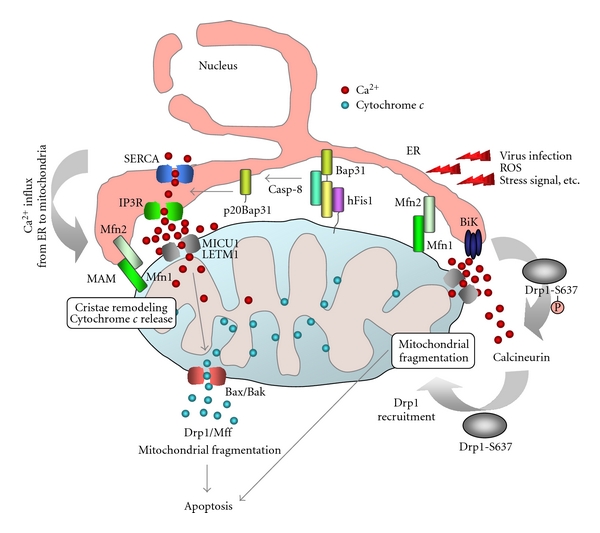
Hypothetical models of the role of contacts between mitochondria and ER in apoptosis. The hFis1/Bap31 platform transmits the mitochondrial stress signal to the ER via the activation of procaspase-8. The cytosolic region of the ER integral membrane protein Bap31 is cleaved by activated caspase-8 to generate proapoptotic p20Bap31, which causes rapid transmission of ER calcium signals to the mitochondria via the IP3 receptor. At close ER-mitochondria contact sites, mitochondria takes up calcium into the matrix via the mitochondrial calcium channels MICU1 or LETM1. The massive influx of calcium leads to mitochondrial fission, cristae remodeling, and cytochrome *c* release. Mfn2 is enriched in the mitochondria-associated membranes (MAM) of the endoplasmic reticulum (ER), where it interacts with Mfn1 and Mfn2 on the mitochondria to form interorganellar bridges. Upon apoptosis signal, a BH3-only member of the Bcl-2 family, Bik, induces Ca^2+^ release from the ER and, in turn, induces Drp1 recruitment to the mitochondria and their fragmentation and cristae remodeling. SERCA, sarco/endoplasmic reticulum Ca^2+^-ATPase. MICU1, mitochondrial calcium uptake 1. LETM1, leucine zipper/EF hand-containing transmembrane 1.

In yeast, the ER mitochondria encounter structure (ERMES comprising cytosolic Mdm12, mitochondrial Mdm10, Mdm34, and Gem1, and ER membrane protein Mmm1), identified by synthetic biology and biochemical approaches [88–90], are involved in phospholipid transport. A similar structure is expected to exist in mammalian cells; a mammalian homolog of yeast Gem1, MIRO, is detected in the proximity of the ER-mitochondria [90]. Ca^2+^ is a key regulator of not only cell survival but also cell death in response to various apoptotic stimuli (Figure 3). The mitochondria and ER have close contacts that are physiologically important for the transfer of Ca^2+^, lipids, and metabolites, and therefore, for the regulation of mitochondrial metabolism and other complex cellular processes including apoptosis. Mfn2 and its regulator Trichoplein/mitostatin localizing on the mitochondria-associated ER membranes (MAM) is involved in tethering mitochondria and ER through hetero- or homotypic interactions with mitochondrial Mfn1 and Mfn2, thereby regulating Ca^2+^ transfer from the ER to the mitochondria [91, 92]. Interactions between the mitochondria and ER are also supported by the finding that the ER can elicit mitochondrial apoptosis. ER targeting of Bik, a BH3-only member of the Bcl-2 family, induces Ca^2+^ release from the ER and its concomitant uptake by the mitochondria, which in turn induces Drp1 recruitment to the mitochondria and their fragmentation and cristae remodeling (Figure 3) [93]. Mammalian mitochondrial Fis1 is an ortholog of yeast Fis1 thought to be involved in recruitment of Drp1 to the mitochondria as in yeast [94]. Although recent experiments revealed that Fis1 is not necessary for Drp1-dependent mitochondrial fission in mammals [10, 24, 25], it might have another important role. In this context, overexpression of hFis1 (for human Fis1) induces mitochondrial fragmentation concomitant with Bax/Bak-dependent release of cytochrome *c* into the cytosol [95]. Of note, hFis1 does not directly activate Bax and Bak, but induces ER Ca^2+^-dependent mitochondrial dysfunction, leading to mitochondrial apoptosis [96]. Interestingly, Iwasawa et al. recently demonstrated that hFis1 localized to the MAM transmits an apoptosis signal from the ER to mitochondria by interacting with Bap31 at the ER to form a platform for the recruitment of the initiator procaspase-8. Apoptotic signals induce cleavage of Bap31 into p20Bap31, which causes the rapid transmission of ER calcium to the mitochondria through inositol triphosphate receptors at the ER-mitochondria junction [97]. Ca^2+^ influx into the mitochondria stimulates Drp1-dependent mitochondrial fission and cytochrome *c* release. Thus, the hFis1-Bap31 complex, bridging the mitochondria and ER, functions as a platform to activate the initiator procaspase in apoptosis signaling (Figure 3). Recently Green and collaborators provided evidence that contacts between mitochondria and other organelles such as ER are involved in regulation of the levels of sphingolipid metabolites that are required for Bax/Bak activation [98]. Distinct from the substrate or ion transfer function of the ER-mitochondria contact, Friedman et al. [99] recently demonstrated that mitochondrial fission occurs at contact regions between the mitochondria and the ER. At the contacts, the ER wraps around the mitochondria to form constrictions, where Drp1 and Mff accumulate and facilitate mitochondrial fission. Interestingly, ER-localized Mfn2 which was shown to be involved in tethering mitochondria and ER [91] is not involved in this reaction [99].

## 8. Conclusions

Although key proteins regulating mammalian mitochondrial dynamics have been identified during the past decade, molecular mechanisms, their coordination, and physiologic functions in distinct tissues are poorly understood, especially in fission reaction: the mechanism of recruitment of Drp1 to fission foci, functional relation between Mff and Mid proteins in the Drp1 recruitment, and regulation of the foci assembly and disassembly. Furthermore, involvement of Fis1 in the regulation of mitochondrial dynamics and its physiologic function remain to be investigated. It is generally agreed, but not completely admitted, that mitochondrial fission/fusion dynamics are related to apoptosis and a balance sift toward fission enhances apoptotic susceptibility of essentially all types of cells. Accumulating evidence, however, suggest that Bax/Bak-dependent MOMP and cytochrome *c* mobilization within IMS after cristae disintegration are essential for cytochrome *c* release and Drp1 facilitates these processes. The mechanisms coordinating these reactions and the effect of Bcl-2 family proteins on mitochondrial fission and fusion machineries remain to be analyzed at the molecular level. Recent studies have revealed that the ER-mitochondria contacts (MAM structures) are involved in the regulation of mitochondrial energy, lipid metabolism, calcium signaling, and even in mitochondrial fission. Identification of additional structural components, regulation of assembly of these structures, and relation between various complexes will reveal novel aspects of cell physiology regulation through communication between mitochondria and ER.

 In conclusion, mitochondrial fusion and fission machineries have crucial function in regulating cell physiology, and investigation of the relation between mitochondrial dynamics and their physiologic function will provide exciting breakthrough in cell biology and various disorders.
